# Association between β2-Adrenoceptor Gene Polymorphisms and Asthma Risk: An Updated Meta-Analysis

**DOI:** 10.1371/journal.pone.0101861

**Published:** 2014-07-03

**Authors:** Haojun Xie, Yuanxiong Cheng, Yating Huo, Guohua Huang, Jin Su

**Affiliations:** Department of Respiratory and Critical Care Medicine, Nanfang Hospital, Southern Medical University, Guangzhou, Guangdong, China; Indiana University Bloomington, United States of America

## Abstract

**Background:**

Evidence is increasingly accumulated about multiple roles for the β2-adrenoceptor gene in asthma. The results were inconsistent partly due to small sample sizes. To assess the association between β2-adrenoceptor gene polymorphisms and asthma risk, a meta-analysis was performed.

**Methods:**

We comprehensively searched the PubMed, EMBASE, BIOSIS Previews databases and extracted data from all eligible articles to estimate the association between β2-adrenoceptor gene polymorphisms and asthma risk. The pooled odds ratio (OR) with 95% confidence intervals (CIs) were calculated.

**Results:**

Thirty-seven studies involving 6648 asthma patients and 15943 controls were included in the meta-analysis. Overall, significant associations were found in allelic genetic model (OR = 1.06, 95% CI = 1.01∼1.12), recessive genetic model (OR = 1.11, 95% CI = 1.02∼1.21) for Arg/Gly16. Stratified by ethnicity and age, significant associations were also found in Asian population in allelic genetic model, recessive genetic model and addictive model. For Gln/Glu27, no significant association was found when we combined all eligible studies. Age stratification showed significant associations in adults in allelic genetic model and recessive genetic model, but no significant association was found among Asians and Caucasians in ethnicity stratification.

**Conclusions:**

This meta-analysis implied that the β2-adrenoceptor Arg/Gly16 polymorphism was likely to contribute to asthma risk in Asian population. Gln/Glu27 polymorphism might be a contributor to asthma susceptibility for adults.

## Introduction

Asthma is a common chronic disorder of the airways, which is characterized by airway hyperresponsiveness, obstruction, and airway wall remodeling [1]. Based on data from World Health Survey, To T and colleagues found that the global prevalence of doctor-diagnosed asthma in adults was estimated at 4.3% [2]. The prevalence reported in several studies indicated that asthma may still be increasing in western countries and developing countries [2]–[4]. Asthma is a complex disease and has a strong genetic component in its pathogenesis. So far, considerable efforts have been made to evaluate the association between genetic variants and asthma risk, and numerous genes have been identified as asthma susceptible genes [5]–[7].

The β2-adrenoceptor (β2AR) mediates the physiological responses in the airway, which include bronchodilation, bronchoprotection, enhanced mucociliary clearance [8]. The β2AR gene is located on chromosome 5q31-q32, a region that is genetically linked to asthma and related phenotypes [9]. There are three best-known polymorphisms in the coding region of the β2AR gene that can modulate the function of the receptor [10]. Many studies have investigated these polymorphisms to assess their potential contributions to the risk of asthma. It was observed in some studies that β2AR gene may play a significant role in the pathogenesis of asthma [11]–[16]. However, other studies showed no association between asthma and β2AR gene polymorphisms [17]–[22]. A large population-based study and three meta-analyses have also shown inconsistent results [23]–[26]. Since then, additional many studies with large sample sizes about β2AR polymorphisms on asthma risk have been reported.

Therefore, we present the results of a comprehensively updated meta-analysis of all relevant published data to investigate the association between β2AR gene polymorphisms and asthma risk with focus on Arg/Gly16 and Gln/Glu27 polymorphism.

## Materials and Methods

### Publication search

We followed criteria of the Preferred Reporting Items for Systematic Reviews and Meta-analysis (PRISMA) to perform this meta-analysis (Dox S1). Two investigators (H.X. and Y.H.) independently carried out a systematic search of the PubMed, EMBASE, BIOSIS Previews databases for articles published until 3 January, 2014 to identify potentially relevant articles. The search strategy utilized in our study were as follows: “asthma or bronchial asthma or bronchial hyperreactivity or allergy or atopy*”* and *“ADRB2* or β2-adrenergic or beta adrenergic or adrenergic or adrenoceptor or beta2 AR*”* and “polymorphism* or variant* or genetic* or mutant*”. No publication language restrictions were imposed. All the searchable studies were retrieved, and we also checked their references for other relevant publications. The detailed search strategy for this study is showed in Dox S2.

### Inclusion and Exclusion Criteria

The inclusion criteria of our study were as follows: (1) Human studies evaluating associations between β_2_-Adrenoceptor gene polymorphisms and asthma risk, (2) There were at least two comparison groups, for example, asthma versus control (nonasthma) group, and (3) Genotype distributions in comparison groups should be available for estimating an odds ratio (OR) with 95% confidence interval (CI). Studies would be discarded if they met one of the following criteria: (1) Not relevant to β_2_-Adrenoceptor polymorphisms or asthma risk, (2) The design based on family or sibling pairs, (3) Reviews or abstracts without useful information. If the same patient population was reported in several publications, we included the most complete study in our meta-analysis. If original genotype frequency data were not reported, we sent an email to the corresponding author for additional data. Studies were excluded from our meta-analysis if their authors did not provide us with related data.

### Data Extraction

In the data collection process, two investigators (H.X. and Y.H.) independently examined full manuscripts of eligible studies, and relevant data were extracted into predesigned data collection form. We verified accuracy of data by comparing collection forms from each investigator. Any disagreement was resolved by discussion, or a third author (Y.C.) would evaluate these articles. The following information was collected from each eligible study: first author's name, year of publication, original country, ethnicity, sample size, genotyping method, age, and genotype numbers in cases and controls.

### Publication Bias

Publication bias of studies was assessed using funnel plot, and P<0.05 was considered statistically significant. In addition, publication bias was also evaluated via Egger’s linear regression test.

### Statistical analysis

We tested the Hardy-Weinberg equilibrium (HWE) in control group using the Chi-square test. The pooled ORs were calculated for allelic genetic model, additive genetic model, dominant genetic model and recessive genetic model, respectively. Subgroup analyses were performed in term of ethnicity and age. The heterogeneity between the studies was investigated by using Cochrane Q-test. I^2^ was also used to test the heterogeneity among the included studies. A p value>0.10 for the Q-test indicates a lack of heterogeneity among the studies, then the pooled OR estimate of each study was calculated by the fixed effect model. Otherwise, the random effect model was used.

Statistical analyses were performed using the Revman5.2 software (Nordic Cochrane Center, Copenhagen, Denmark), STATA 12.0 software (Stata Corporation, College Station, TX). A p value<0.05 was considered statistically significant, except for test of heterogeneity where a level of p value<0.10 was used.

## Results

### Characteristics of studies included in the Meta-analysis

**Figure 1:**
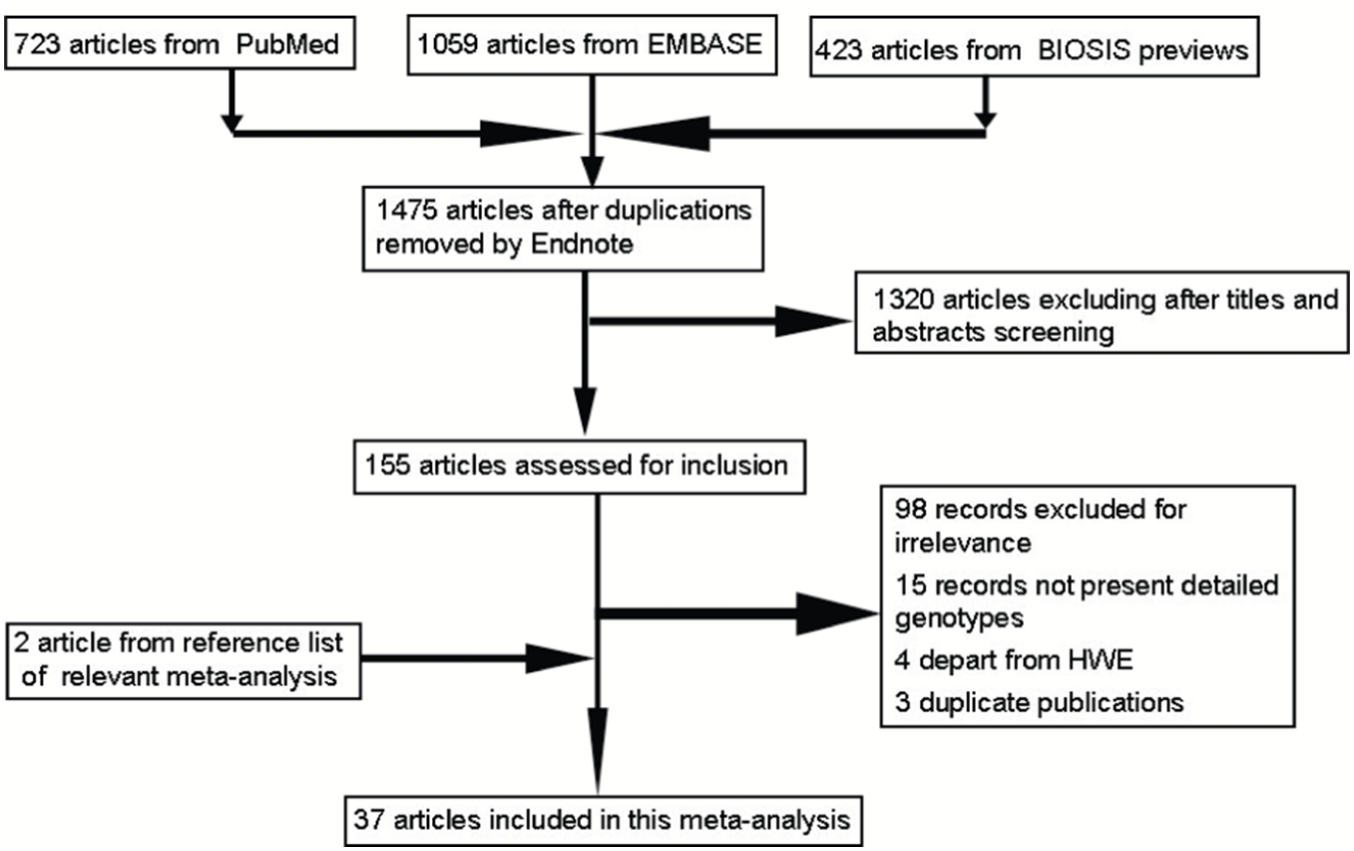
Flow Diagram for Inclusion of Studies in Meta-analysis. The initial search identified 2205 articles, of which, 37 were included in the final analysis.

The flow chart in Figure 1 outlined the study selection process. A total of 2205 articles were identified at the initial search. 730 duplicated studies were eliminated first. After reading the abstracts and titles, 1320 articles were subsequently excluded. The remaining 155 articles were then assessed for inclusion and 120 articles were excluded (98 records excluded for irrelevance, 15 records no detailed genotypes, 4 depart from HWE, 3 duplicate publications). In this process, study references were checked as well and 2 relevant publication were identified [20], [27]. Finally, a total of 37 studies met the inclusion criteria, and were included in the meta-analysis [11]–[22], . These eligible studies contained 6648 asthma cases and 15943 controls, and 11 Caucasian, 18 Asian and 8 other populations. The distributions of the β2AR gene polymorphism in normal controls were consistent with HWE in every study. Details of each study included in this meta-analysis were summarized in Table 1.

**Table 1:** Characteristics of studies included in meta-analysis.

First author[Ref]	Year	Country	Ethnicity	Case (n)	Control (n)	Study design	Age group	GenotypingMethod
Abdul Vahab Saadi#	2013	India	Asian	150	150	Case-control	Mixed	Sequencing
Al-Rubaish A#	2011	Saudi Arabia	Arabia	73	85	Case-control	Children	PCR-RFLP
Barr RG#	2001	USA	Caucasian	171	137	Case-control	Adults	Allele specific
Bhatnagar, P #	2005	India	Asian	101	55	Case-control	Adults	Sequencing
Binaei S#	2003	USA	Caucasian	38	155	Case-control	Children	PCR-RFLP
Birbian N#	2012	India	Asian	410	414	Case-control	Adults	PCR-RFLP
Chan IH#	2008	China	Asian	295	173	Case-control	Children	PCR-RFLP
ChiangCH#	2012	China	Asian	476	115	Case-control	Adults	PCR-RFLP
Dewar JC#	1998	UK	Caucasian	117	511	Cross-section	Adults	Allele specific
Gao G#	2000	China	Asian	58	89	Case-control	Adults	PCR-RFLP
Gao JM#	2002	China	Asian	125	96	Case-control	Adults	PCR-RFLP
Hakonarson H#	2001	Iceland	Caucasian	324	199	Case-control	Adults	Sequencing
Holloway JW#	2000	New Zealand	Caucasian	154	91	Case-control	Adults	Sequencing
Hopes E#	1998	UK	Caucasian	102	317	Cross-section	Children	Allele specific
Isaza C#	2012	Colombia	NA	109	137	Case-control	Children	Sequencing
Karam RA#	2013	Egypt	African	90	110	Case-control	Children	Allele specific
Kohyama K#	2011	Japan	Asian	300	100	Case-control	Adults	Sequencing
Kotani Y#	1999	Japan	Asian	117	103	Case-control	Adults	Allele specific
Leung TF#	2002	China	Asian	76	70	Case-control	Children	PCR-RFLP
Li H#	2009	China	Asian	192	192		Children	PCR-RFLP
Lin YC#	2003	China	Asian	80	69	Case-control	Children	Allele specific
Martinez FD#	1997	USA	Caucasian	38	229	Cross-section	Children	PCR-RFLP
Matheson MC#	2006	Australia	Caucasian	123	221	Case-control	Adults	ARMS
Munakata M#	2006	Japan	Asian	46	100	Case-control	NA	Sequencing
Pino-Yanes M#	2013	Spain	Hispanic	596	1240	Case-control	Children	iPLEXH Gold assay
Potter PC#	1993	South Africa	African	22	30	Case-control	Children	PCR-RFLP
Qiu YY#	2010	China	Asian	201	276	Case-control	Adults	Sequencing
Reihsaus E#	1993	USA	NA	51	56	Case-control	Adults	Sequencing
Santillan AA#	2003	Mexico	NA	303	604	Case-control	Adults	PCR-RFLP
Shachor J#	2003	Israel	Arabia	66	113	Case-control	Adults	PCR-RFLP
Szczepankiewicz A#	2009	Poland	Caucasian	113	121	Case-control	Children	PCR-RFLP
Tatarskyy, P.F #	2011	Ukraine	Caucasian	114	86	Case-control	Children	Multiplexed PCR
Thomsen M#	2012	Denmark	Caucasian	547	8386	Cross-section	Adults	PCR-RFLP
Wang JY#	2009	China	Asian	442	545	Case-control	Children	Taq Man
Wang Z#	2001	China	Asian	128	136	Case-control	Adults	Allele specific
Ye, Y.M#	2010	Korea	Asian	102	322	Case-control	Adults	Sequencing
Zheng, B.Q#	2012	China	Asian	198	110	Case-control	Children	Sequencing

NA, not available; PCR, polymerase chain reaction; RFLP, restricted fragment length polymorphisms; SSP, sequence.

-specific primers; SPT, skin prick test; BHR,bronchial hyperresponsiveness; TDI, toluene diisocyanate; ARMS, amplification refractory mutation system;Mixed, participants involved children and adults.

### β2AR Arg/Gly16 and Asthma Risk

Among 37 studies included in the meta-analysis, 29 investigated the contribution of β2AR Arg/Gly16 polymorphism to asthma risk (Table S1). Overall, slightly significant associations were found in allelic genetic model (OR = 1.06, 95% CI = 1.01∼1.12, p = 0.02), additive genetic model (OR = 1.11, 95% CI = 1.02∼1.21, p = 0.02) for Arg/Gly16. Stratified by ethnicity, significant associations were also found in Asian population in allelic genetic model (OR = 1.14, 95% CI = 1.05∼1.23, p = 0.001), recessive genetic model (OR = 1.26, 95% CI = 1.12∼1.43, p = 008) and additive model (OR = 1.23, 95% CI = 1.06∼1.44, p = 0001), but not in Caucasian population. No association was found in adults and children subgroup in term of age (Table 2).

**Table 2:** Results of the pooled analyses and subgroup analyses for the β2AR Arg/Gly16 polymorphism and asthma risk.

Variable	n	Case/Control	Arg16 vs Gly16			Arg16Arg vs Gly16Gly			Arg16Arg vs Gly16Gly/ Arg16Gly			Gly16Gly vs Arg16Arg/ Arg16Gly		
			OR[95%CI] (p)	P-het	I^2^	OR[95%CI] (p)	P-het	I^2^	OR[95%CI] (p)	P-het	I^2^	OR[95%CI] (p)	P-het	I^2^
Overall	29	5595/15184	1.06[1.01,1.12] **0.02**	<0.00001	64%	1.11[1.02,1.21] **0.02**	0.0007	52%	0.94[0.87,1.02] 0.12	0.0006	52%	1.10[0.99,1.22] 0.06	<0.0001	58%
Ethnicity														
Asian	15	2882/2842	1.14[1.05,1.23] **0.001**	<0.0001	67%	1.26[1.12,1.43] **0.0001**	0.008	53%	0.91[0.81,1.04] 0.16	0.005	55%	1.23[1.06,1.44] **0.008**	0.0002	66%
Caucasiann	10	1726/10355	0.99[0.91,1.08] 0.89	0.002	65%	0.94[0.80,1.10] 0.45	0.09	40%	0.98[0.87,1.10] 0.72	0.003	64%	0.98[0.82,1.17] 0.81	0.03	52%
Age														
Children	10	1817/3009	1.05[0.97,1.15] 0.23	0.0005	70%	1.11[0.96,1.28] 0.15	0.005	62%	0.96[0.84,1.10] 0.58	0.010	59%	1.06[0.89,1.26] 0.52	0.002	66%
Adults	17	3582/11925	1.04[0.98,1.11] 0.22	0.003	55%	1.06[0.95,1.18] 0.32	0.08	35%	0.95[0.86,1.05] 0.31	0.01	50%	1.07[0.94,1.22] 0.32	0.02	47%

n, number of studies.

### β2AR Gln/Glu27 and Asthma Risk

There were 22 studies investigating the association between Gln/Glu27 polymorphism and asthma risk (Table S2). One study [32] may be the main contributor to heterogeneity and has significantly influenced pooled results (discuss below). Therefore, it was not included in data synthesis process. Overall, no significant association was found in all genetic models when combined all other 21 studies (Table 3). Age stratification showed significant associations in adults in allelic genetic model (OR = 1.10, 95% CI = 1.01∼1.19, p = 0.03) and additive genetic model (OR = 1.20, 95% CI = 1.07∼1.34, p = 0.002), but no association was found in ethnicity stratification (Table 3).

**Table 3:** Results of the pooled analyses and subgroup analyses for the β2AR Gln/Glu27 polymorphism and asthma risk.

Variable	n	Case/Control	Gln27 vs Glu27			Gln27Gln vs Glu27Glu			Gln27Gln vs Glu27Glu/ Gln27Glu			Glu27Glu vs Gln27Gln/ Gln27Glu		
			OR[95%CI] (p)	P-het	I^2^	OR[95%CI] (p)	P-het	I^2^	OR[95%CI] (p)	P-het	I^2^	OR[95%CI](p)	P-het	I^2^
Overall	22	3363/12337	1.07[0.99,1.15] 0.09	0.03	39%	1.11[1.01,1.23] 0.03	0.005	49%	1.02[0.89,1.18] 0.77	0.64	0%	1.02[0.87,1.20] 0.80	0.89	0%
Overall#	21	3060/11733	1.02[0.95,1.10] 0.59	0.50	0%	1.03[0.93,1.14] 0.60	0.34	9%	1.02[0.89,1.18] 0.74	0.58	0%	1.01[0.86,1.20] 0.86	0.86	0%
Ethnicity														
Asian	9	1306/1286	1.10[0.91,1.32] 0.34	0.37	8%	1.18[0.96,1.46] 0.18	0.32	14%	0.72[0.42,1.23] 0.23	0.82	0%	0.75[0.43,1.30] 0.31	0.93	0%
Caucasiann	7	1367/9948	1.03[0.94,1.12] 0.58	0.20	30%	1.02[0.89,1.17] 0.12	0.38	7%	1.05[0.90,1.24] 0.51	0.10	44%	1.06[0.88,1.28] 0.51	0.25	23%
Age														
Children	8	682/1104	0.96[0.81,1.13] 0.63	0.65	0%	0.86[0.69,1.07] 0.18	0.79	0%	1.21[0.86,1.73] 0.28	0.56	0%	1.00[0.68,1.47] 0.99	0.76	0%
Adults	12	2485/10987	1.10[1.01,1.19] **0.03**	0.007	57%	1.20[1.07,1.34] 0.002	**0.002**	62%	1.01[0.86,1.18] 0.93	0.57	0%	1.05[0.88,1.26] 0.59	0.66	0%

# Pooled result not included study of Santilan, A A#, n, number of studies.

### Heterogeneity exploration and sensitivity analysis

**Figure 2:**
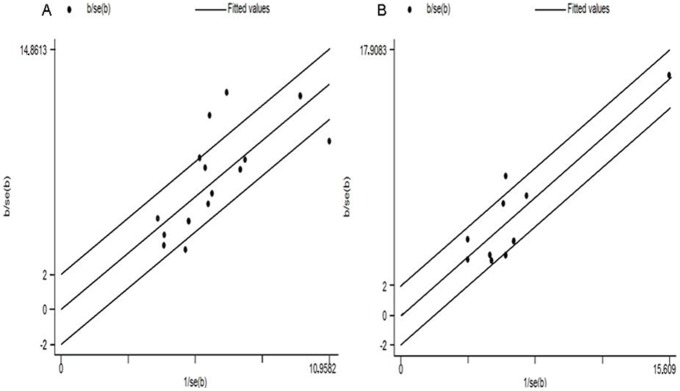
Galbraith plots of the association between β2AR Arg16Gly polymorphism and asthma. A) Asian population subgroup; B) caucasian population subgroup. Four studies# maybe the main contributors to the heterogeneity Asian population subgroup; One studies# maybe the main contributors to the heterogeneity Asian population subgroup.

For β2AR Arg/Gly16 polymorphism, there were statistically significant heterogeneities in all genetic models when all eligible studies were combined (Table 2). We thus performed subgroup analysis on the basis of age and ethnicity. Statistically significant heterogeneity remained shown in Asian and Caucasian population subgroup, children subgroup and adults subgroup (Table 2). Therefore,we used Galbraith plots to graphically evaluate the source of heterogeneity. As showed in Figure 2A, four studies [31], [35], [47], [50] may be the main contributor to the heterogeneity in Asian population subgroup, and Figure 2B indicated that one study [15] maybe the main contributors to the heterogeneity in Caucasian population subgroup. In order to assess the reliablity of our meta-analysis, we further performed a sensitivity analysis through sequentially excluded individual study and similar result was obtained.

For β2AR Gln/Glu27 polymorphism, statistically significant heterogeneity were found in all genetic models, but quantitatively moderate heterogeneity with I^2^ values<50% (Table 3). Sensitivity analysis through sequentially excluded individual study shown that one study [32] maybe the main contributor to heterogeneity and has significantly influenced pooled results. Combined results excluding this study showed small heterogeneity for both Asian and Caucasian population (Table 3).

### Publication bias

**Figure 3:**
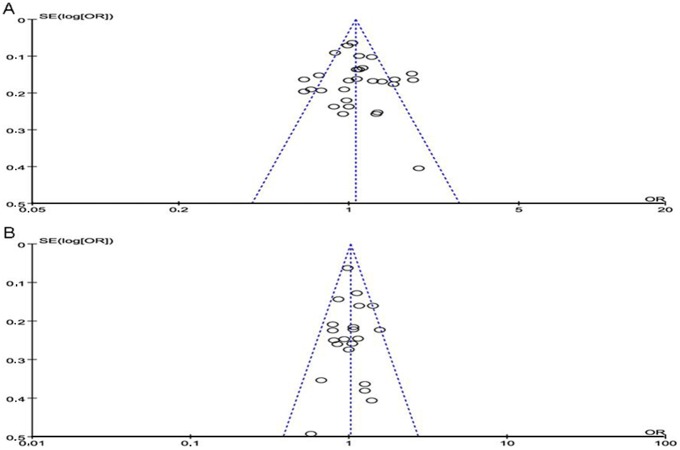
Funnel plot of publication bias for β2AR polymorphism under recessive genetic model. A) Arg/Gly16; B) Gln/Glu27. Funnel plot with pseudo 95% confidence limits was used.

For β2AR Arg/Gly16 polymorphism, as shown in Figure 3A, the shape of funnel plot revealed asymmetry to some extent. For β2AR Gln/Glu27 polymorphism, the shape of funnel plot did not indicate any evidence of significant asymmetry (Figure 3B). However, the Egger’s test yielded no evidence of publication bias for β2AR Arg/Gly16 polymorphism (A vs G, p = 0.357), and Gln/Glu27 polymorphism (A vs G, p = 0.764).

## Discussion

Asthma is a public health problem, affecting approximately 300 million people worldwide [52]. The mainstay of treatment for acute asthma includes inhaled short-acting β2-adrenergic receptor (β2AR) agonists, such as albuterol [5], [53]. There is mounting evidence that polymorphisms in the β2AR gene are associated with significant variability in response to short-acting β2-agonists (SABA). In adults, genetic variations in this receptor have also been linked to asthma severity [12], [54]. In children, significant association was observed between favorable therapeutic response to inhaled β2-adrenergic agonists and the Arg/Arg phenotype at position 16 of the β2AR [55]. However, a large cohort study has shown that important β2AR gene polymorphisms are not main determinants of asthma incidence or prevalence in the British population [25]. Therefore, conflicting data regarding the role of β2AR polymorphisms in asthma susceptibility and presentation has been reported.

We performed a systematic review and meta-analysis, including 37 studies involving 6648 asthma patients and 15943 controls, to investigate the associations between β2AR Arg/Gly16 and Gln/Glu27 polymorphisms and asthma. The combined results of 29 studies showed that A allele carriers of Arg/Gly16 polymorphism is strongly associated with asthma risk in the total population. Stratification by ethnicity revealed statistically significant association in the Asian population. Nonetheless, overall we failed to find any association between Gln/Glu27 polymorphisms and asthma risk for 21 eligible studies. Subgroup analysis on the basis of age and ethnicity showed significant associations in adults in allelic genetic model and recessive genetic model for Gln/Glu27. However, no association was found in Asian and Caucasian population subgroup based on ethnicity under any genetic model. Sensitivity analyses through sequentially excluding individual study yielded similar results in both β2AR Arg/Gly16 and Gln/Glu27 polymorphisms. In our study, we could not assess haplotype effects, which would have required genotype data that were unavailable in the published studies.

So far, three previous meta-analyses have been conducted to explore the function of β2AR gene polymorphisms in asthma [23], [24], [26]. One of them shown that the Gly16 allele of the β2-adrenergic receptor gene increases the risk of nocturnal asthma, but does not alter the risk of mild asthma [24]. The other reported that Gly16 may have a protective effect for children and Glu27 carriers may decrease the risk of asthma [23]. The third one showed that ADRB2 does not contribute to susceptibility of asthma [26]. Interestingly, our meta-analysis results are different from those of published meta-analysises, showing that Arg16 may increased the risk of asthma in total population, especially for Asian population. For Gln/Glu27, significant associations could only be found in adults in allelic genetic model and recessive genetic model. The inconsistency between the results of ours and published articles may be partly attributed to the following factors. First of all, while in our meta-analysis 37 studies (29 for Arg/Gly16 and 21 for Gln/Glu27, respectively) involving 6648 asthma patients and 15493 controls were included, Migita,O [26] included 8 studies for Arg/Gly16 and 9 for Gln/Glu27, Thakkinstian,A and colleagues [23] only included 12 studies for Arg/Gly16 and 12 for Gln/Glu27, Contopoulos-Ioannidis,D.G [24] included 17 studies for Arg/Gly16 and 16 for Gln/Glu27 respectively. Second, some articles departing from HWE have been included in Contopoulos-Ioannidis D. G and Migita, O^,^s meta-analysis [24], [26]. Deviation from HWE is taken as an indication that the alleles are not segregating independently, either for genetic reasons or methodological reasons [56]. Therefore, this may be a potential source of inconsistency between our study and other meta-analyses.

Genome-wide association study (GWAS) has been productive genotyping method to test a vast number of single nucleotide polymorphisms and assess their relations with complex diseases and phenotypes [57]. Since the first GWAS investigating susceptibility gene for asthma published in 2007 [58], more than ten GWAS of asthma have been performed in Caucasian, Mexican, and African-ancestry populations [51], [59], [60] and more recently in Japan and Singapore of Asian populations [61]–[63]. Nevertheless, neither an individual GWAS study nor meta-analysis of GWAS has implicated ADRB2 the susceptibility gene for asthma. In our meta-analysis, we found that neither ADRB2 Arg/Gly16 nor ADRB2 Gln/Glu27 has significant association with asthma in Caucasian population, which further confirmed the results of previous published single GWAS and meta-analysis [51], [59], [60]. However, significant associations were found between ADRB2 Arg/Gly16 and asthma in Asian population, and this result contrasts with the results of studies recently conducted in Japan and Singapore [61]–[63], in which no single nucleotide polymorphisms in ADRB2 gene was found statistically significant. To date, GWAS studies for asthma have not been conducted in Chinese subjects, we are not sure whether the genomic findings of recent studies are applicable to Chinese. Therefore, in order to further clarify the role of ADRB2 gene in the pathogenesis of asthma, in the future, more GWAS should be conducted in Asian population, and especially Chinese considering its enormous population.

Several strengths of this meta-analysis could be listed as follows. First, we followed a rigorous protocol of systematic review, identifying data from 3 different databases comprehensively. Second, we have conducted subgroup analysis, sensitivity analysis and Galbraith plots to explore the source of heterogeneity, suggesting the reliability of our study. However, the results of our meta-analysis should be interpreted with caution due to some unavoidable limitations. First, potential heterogeneity and confounding factors may have affected the analysis. Statistically significant and quantitatively moderate–to–high heterogeneities were found in all genetic model for Arg/Gly16, even if we have found out the potential heterogeneity contributors. Second, publication bias might result in the loss of studies, which subsequently could affect the meta-analysis results. The shapes of funnel plots revealed slight asymmetry for Arg/Gly16 and we should pay attention to this point. Third, asthma is a heterogeneous group of conditions that result in recurrent, reversible bronchial obstruction [5]. As shown in Table 1 that the asthma definition varied among different articles and this may be a confounding factor. Case–control studies with clear diagnosis criteria and rigorous quality control are needed to conducted in the future. Fourth, most of the studies included in our meta-analysis were performed in Asians and Caucasians. Thus, our results may be applicable only to these ethnic groups. Fifth, although we have tried our best to contact the corresponding authors of published original papers and meta-analysis, we still cannot gain genotypes data of several studies. Sixth, so many tests have been conducted in our meta-analysis that it is not sure whether we have found something significant by chance in this process. Finally, asthma itself is a complex and multifactorial disease and potential interactions among gene-gene and gene-environment should be considered.

## Conclusion

The present meta-analysis suggests that β2AR Arg/Gly16 polymorphism may be an important genetic factor in the overall risk for developing asthma, especially in Asian population, and Gln/Glu27 polymorphisms may be a contributor to asthma susceptibility for adults. GWAS or more case–control studies with explicit diagnosis criteria and rigorous quality control are needed to support this finding in various ethnic groups.

## Supporting Information

Table S1 Distribution of *Arg/Gly*16 genotypes among patients with asthma and controls included in the meta-analysis.
(DOC)

Table S2 Distribution of Gln/Gln27genotypes among patients with asthma and controls included in the meta-analysis.
(DOC)

Dox S1 PRISMA 2009 Statement checklist.
(DOC)

Dox S2 Search strategies for this study.
(DOC)
