# Supplementary material for: Association between β2-Adrenoceptor Gene Polymorphisms and Asthma Risk: An Updated Meta-Analysis
Source: PLoS One. 2014 Jul 3;9(7):e101861. doi: 10.1371/journal.pone.0101861 (PMC4081822; doi:10.1371/journal.pone.0101861)
Supplement: Table S1 — Distribution of Arg/Gly16 genotypes among patients with asthma and controls included in the meta-analysis. (DOC) [file pone.0101861.s001.doc]

**TableS1**. Distribution of *Arg/Gly*16 genotypes among patients with asthma and controls included in the meta-analysis.

| First author | Asthma | | |  | Control | | | Hardy–Weinberg equilibrium |
| --- | --- | --- | --- | --- | --- | --- | --- | --- |
|  | *ArgArg16* | *Ar/Gly16* | *GlyGly16* |  | *ArgArg16* | *ArgGly16* | *GlyGly16* |  |
| AbdulVahab Saadi[47] | 58 | 57 | 35 |  | 31 | 66 | 53 | 0.217 |
| Barr RG[30] | 36 | 97 | 38 |  | 51 | 62 | 24 | 0.495 |
| Bhatnagar,P[16] | 19 | 54 | 28 |  | 12 | 30 | 13 | 0.498 |
| Binaei S[48] | 7 | 24 | 7 |  | 34 | 67 | 54 | 0.132 |
| Birbian N[41] | 62 | 199 | 149 |  | 48 | 188 | 178 | 0.877 |
| Chan IH[27] | 101 | 135 | 59 |  | 51 | 89 | 33 | 0.597 |
| Dewar JC[19] | 14 | 50 | 53 |  | 74 | 263 | 180 | 0.158 |
| Gao JM[31] | 38 | 59 | 28 |  | 35 | 53 | 8 | 0.051 |
| Hakonarson H[20] | 45 | 151 | 127 |  | 21 | 85 | 75 | 0.676 |
| Holloway JW[12] | 29 | 47 | 78 |  | 17 | 39 | 35 | 0.303 |
| Hopes E[29] | 11 | 54 | 37 |  | 28 | 147 | 142 | 0.245 |
| Kohyama K[40] | 40 | 160 | 100 |  | 15 | 50 | 35 | 0.676 |
| Kotani Y[11] | 30 | 52 | 35 |  | 28 | 45 | 30 | 0.201 |
| Leung TF[21] | 25 | 38 | 13 |  | 22 | 37 | 11 | 0.482 |
| Li H[35] | 86 | 76 | 30 |  | 46 | 100 | 46 | 0.563 |
| Martinez FD[28] | 5 | 18 | 15 |  | 33 | 108 | 88 | 0.988 |
| Matheson MC[15] | 18 | 69 | 36 |  | 21 | 102 | 98 | 0.451 |
| Munakata M[34] | 14 | 21 | 11 |  | 23 | 47 | 30 | 0.579 |
| Pino-Yanes M[51] | 104 | 286 | 206 |  | 228 | 576 | 436 | 0.121 |
| Potter PC[17] | 9 | 9 | 4 |  | 6 | 15 | 9 | 0.956 |
| Qiu YY[37] | 77 | 85 | 39 |  | 88 | 135 | 53 | 0.923 |
| Santillan AA[32] | 56 | 163 | 84 |  | 101 | 318 | 185 | 0.156 |
| Shachor J[33] | 11 | 38 | 17 |  | 26 | 52 | 35 | 0.433 |
| Szczepankiewicz A[36] | 16 | 48 | 49 |  | 26 | 54 | 41 | 0.303 |
| Thomsen M[44] | 84 | 256 | 207 |  | 1243 | 3866 | 3277 | 0.062 |
| Wang JY[50] | 138 | 207 | 97 |  | 173 | 250 | 87 | 0.837 |
| Wang Z[14] | 52 | 54 | 22 |  | 38 | 64 | 34 | 0.499 |
| Ye,Y.M[38] | 30 | 53 | 18 |  | 98 | 153 | 71 | 0.441 |
| Zheng, B.Q[45] | 71 | 99 | 28 |  | 31 | 55 | 24 | 0.966 |
