# Supplementary material for: Association between β2-Adrenoceptor Gene Polymorphisms and Asthma Risk: An Updated Meta-Analysis
Source: PLoS One. 2014 Jul 3;9(7):e101861. doi: 10.1371/journal.pone.0101861 (PMC4081822; doi:10.1371/journal.pone.0101861)
Supplement: Table S2 — Distribution of Gln/Gln27genotypes among patients with asthma and controls included in the meta-analysis. (DOC) [file pone.0101861.s002.doc]

**TableS2**. Distribution of Gln/Gln27genotypes among patients with asthma and controls included in the meta-analysis.

| First author | Asthma | | |  | Control | | | Hardy–Weinberg equilibrium |
| --- | --- | --- | --- | --- | --- | --- | --- | --- |
|  | GlnGln27 | GlnGlu27 | GluGlu27 |  | GlnGln27 | GlnGlu27 | GluGlu27 |  |
| Abdul Vahab Saadi[47] | 112 | 26 | 12 |  | 104 | 39 | 7 | 0.193 |
| Al-Rubaish A[39] | 41 | 25 | 7 |  | 50 | 30 | 5 | 0.859 |
| Chiang CH[42] | 400 | 66 | 10 |  | 85 | 29 | 1 | 0.384 |
| Dewar JC[19] | 33 | 51 | 35 |  | 134 | 271 | 106 | 0.148 |
| Gao G[13] | 20 | 32 | 6 |  | 32 | 49 | 8 | 0.171 |
| Hakonarson H[20] | 92 | 173 | 59 |  | 48 | 112 | 39 | 0.071 |
| Hopes E[29] | 24 | 63 | 15 |  | 83 | 156 | 78 | 0.782 |
| Isaza C[43] | 76 | 29 | 4 |  | 103 | 29 | 5 | 0.119 |
| Karam RA[46] | 44 | 38 | 8 |  | 64 | 36 | 10 | 0.149 |
| Kotani Y[11] | 94 | 23 | 0 |  | 89 | 14 | 0 | 0.459 |
| Leung TF[21] | 64 | 12 | 0 |  | 55 | 15 | 0 | 0.315 |
| Lin YC[22] | 65 | 15 | 0 |  | 54 | 14 | 1 | 0.932 |
| Martinez FD[28] | 16 | 17 | 5 |  | 94 | 104 | 32 | 0.707 |
| Matheson MC[15] | 46 | 56 | 21 |  | 59 | 109 | 53 | 0.849 |
| Munakata M[34] | 39 | 6 | 1 |  | 86 | 10 | 0 | 0.590 |
| Qiu YY[37] | 166 | 32 | 3 |  | 226 | 45 | 5 | 0.129 |
| Reihsaus E[18] | 13 | 26 | 12 |  | 17 | 23 | 16 | 0.182 |
| Santillan AA[32] | 241 | 53 | 9 |  | 385 | 202 | 17 | 0.117 |
| Shachor J[33] | 33 | 27 | 4 |  | 53 | 49 | 9 | 0.617 |
| Tatarskyy,P.F[49] | 40 | 56 | 18 |  | 38 | 36 | 12 | 0.466 |
| Thomsen M[44] | 169 | 273 | 105 |  | 2675 | 4062 | 1647 | 0.137 |
| Ye,Y.M[38] | 93 | 8 | 1 |  | 280 | 37 | 1 | 0.849 |
