# Supplementary material for: Association between β2-Adrenoceptor Gene Polymorphisms and Asthma Risk: An Updated Meta-Analysis
Source: PLoS One. 2014 Jul 3;9(7):e101861. doi: 10.1371/journal.pone.0101861 (PMC4081822; doi:10.1371/journal.pone.0101861)
Supplement: Dox S2 — Search strategies for this study. (DOC) [file pone.0101861.s004.doc]

**Search strategy for meta-analysis of association between beta2AR gene polymorphisms and the risk of asthma**

**PubMed**

#1.“polymorphism, genetic”[MH] OR “polymorphism, single nucleotide”[MH]

#2.“polymorphism*”[ALL] OR “variant*”[ALL] OR “genetic* ”[ALL] OR “mutant*”[ALL]

#3. #1 OR #2

#4. “asthma”[MH]

# 5.“asthma”[ALL] OR “bronchial hyperreactivity” [ALL] OR “respiratory hypersensitivity” [ALL] OR allergy[ALL] OR atopy[ALL]

#6. #4 OR #5

#7. adrenergic[ALL] OR β2-adrenergic[ALL] OR beta adrenergic[ALL] OR beta2AR[ALL] OR adrenoceptor[ALL] OR ADRB2[ALL]

#8. #3 AND #6 AND #7

MH = MeSH terms, ALL = All fields

Timespan=1865-2014

Date of Search: Jan. 03 .2014

Results: **723** articles were found

**EMBASE**

- [View results](http://211.103.242.218/GW2/s/com/embase/www/G.http/search/results?viewsearch=20)
- [Edit](http://211.103.242.218/GW2/s/com/embase/www/G.http/search/results)
- [Copy to advanced search](http://211.103.242.218/GW2/s/com/embase/www/G.http/search/advanced?id=20)
- [Set email alert](http://211.103.242.218/GW2/s/com/embase/www/G.http/search/results)
- [Set RSS feed](http://211.103.242.218/GW2/s/com/embase/www/G.http/search/results)
- [View results](http://211.103.242.218/GW2/s/com/embase/www/G.http/search/results?viewsearch=18)
- [Edit](http://211.103.242.218/GW2/s/com/embase/www/G.http/search/results)
- [Copy to advanced search](http://211.103.242.218/GW2/s/com/embase/www/G.http/search/advanced?id=18)
- [Set email alert](http://211.103.242.218/GW2/s/com/embase/www/G.http/search/results)
- [Set RSS feed](http://211.103.242.218/GW2/s/com/embase/www/G.http/search/results)
- [View results](http://211.103.242.218/GW2/s/com/embase/www/G.http/search/results?viewsearch=15)
- [Edit](http://211.103.242.218/GW2/s/com/embase/www/G.http/search/results)
- [Copy to advanced search](http://211.103.242.218/GW2/s/com/embase/www/G.http/search/advanced?id=15)
- [Set email alert](http://211.103.242.218/GW2/s/com/embase/www/G.http/search/results)
- [Set RSS feed](http://211.103.242.218/GW2/s/com/embase/www/G.http/search/results)
- [View results](http://211.103.242.218/GW2/s/com/embase/www/G.http/search/results?viewsearch=12)
- [Edit](http://211.103.242.218/GW2/s/com/embase/www/G.http/search/results)
- [Copy to advanced search](http://211.103.242.218/GW2/s/com/embase/www/G.http/search/advanced?id=12)
- [Set email alert](http://211.103.242.218/GW2/s/com/embase/www/G.http/search/results)
- [Set RSS feed](http://211.103.242.218/GW2/s/com/embase/www/G.http/search/results)
- [View results](http://211.103.242.218/GW2/s/com/embase/www/G.http/search/results?viewsearch=11)
- [Edit](http://211.103.242.218/GW2/s/com/embase/www/G.http/search/results)
- [Copy to advanced search](http://211.103.242.218/GW2/s/com/embase/www/G.http/search/advanced?id=11)
- [Set email alert](http://211.103.242.218/GW2/s/com/embase/www/G.http/search/results)
- [Set RSS feed](http://211.103.242.218/GW2/s/com/embase/www/G.http/search/results)
- [View results](http://211.103.242.218/GW2/s/com/embase/www/G.http/search/results?viewsearch=7)
- [Edit](http://211.103.242.218/GW2/s/com/embase/www/G.http/search/results)
- [Copy to advanced search](http://211.103.242.218/GW2/s/com/embase/www/G.http/search/advanced?id=7)
- [Set email alert](http://211.103.242.218/GW2/s/com/embase/www/G.http/search/results)
- [Set RSS feed](http://211.103.242.218/GW2/s/com/embase/www/G.http/search/results)
- [View results](http://211.103.242.218/GW2/s/com/embase/www/G.http/search/results?viewsearch=6)
- [Edit](http://211.103.242.218/GW2/s/com/embase/www/G.http/search/results)
- [Copy to advanced search](http://211.103.242.218/GW2/s/com/embase/www/G.http/search/advanced?id=6)
- [Set email alert](http://211.103.242.218/GW2/s/com/embase/www/G.http/search/results)
- [Set RSS feed](http://211.103.242.218/GW2/s/com/embase/www/G.http/search/results)
- [View results](http://211.103.242.218/GW2/s/com/embase/www/G.http/search/results?viewsearch=5)
- [Edit](http://211.103.242.218/GW2/s/com/embase/www/G.http/search/results)
- [Copy to advanced search](http://211.103.242.218/GW2/s/com/embase/www/G.http/search/advanced?id=5)
- [Set email alert](http://211.103.242.218/GW2/s/com/embase/www/G.http/search/results)
- [Set RSS feed](http://211.103.242.218/GW2/s/com/embase/www/G.http/search/results)
- [View results](http://211.103.242.218/GW2/s/com/embase/www/G.http/search/results?viewsearch=4)
- [Edit](http://211.103.242.218/GW2/s/com/embase/www/G.http/search/results)
- [Copy to advanced search](http://211.103.242.218/GW2/s/com/embase/www/G.http/search/advanced?id=4)
- [Set email alert](http://211.103.242.218/GW2/s/com/embase/www/G.http/search/results)
- [Set RSS feed](http://211.103.242.218/GW2/s/com/embase/www/G.http/search/results)
- [View results](http://211.103.242.218/GW2/s/com/embase/www/G.http/search/results?viewsearch=3)
- [Edit](http://211.103.242.218/GW2/s/com/embase/www/G.http/search/results)
- [Copy to advanced search](http://211.103.242.218/GW2/s/com/embase/www/G.http/search/advanced?id=3)
- [Set email alert](http://211.103.242.218/GW2/s/com/embase/www/G.http/search/results)
- [Set RSS feed](http://211.103.242.218/GW2/s/com/embase/www/G.http/search/results)
- [View results](http://211.103.242.218/GW2/s/com/embase/www/G.http/search/results?viewsearch=2)
- [Edit](http://211.103.242.218/GW2/s/com/embase/www/G.http/search/results)
- [Copy to advanced search](http://211.103.242.218/GW2/s/com/embase/www/G.http/search/advanced?id=2)
- [Set email alert](http://211.103.242.218/GW2/s/com/embase/www/G.http/search/results)
- [Set RSS feed](http://211.103.242.218/GW2/s/com/embase/www/G.http/search/results)

#1. polymorphism [EXT]

#2. variant* [EXT]

#3. genetic* [EXT]

#4. mutant* [EXT]

#5.“single nucleotide” [EXT]

#6. #1 OR #2 OR #3 OR #4 OR #5

#7. 'asthma'/exp OR asthma

#8. 'allergy'/exp OR allergy

#9. 'atopy'/exp OR atopy

#10.#7 OR #8 OR #9

#11. 'adrenoceptor'/exp OR adrenoceptor

#12. 'β2 adrenergic' [EXT]

- [View results](http://211.103.242.218/GW2/s/com/embase/www/G.http/search/results?viewsearch=22)
- [Edit](http://211.103.242.218/GW2/s/com/embase/www/G.http/search/results)
- [Copy to advanced search](http://211.103.242.218/GW2/s/com/embase/www/G.http/search/advanced?id=22)
- [Set email alert](http://211.103.242.218/GW2/s/com/embase/www/G.http/search/results)
- [Set RSS feed](http://211.103.242.218/GW2/s/com/embase/www/G.http/search/results)

#13. 'ADRB2'[EXT]

#14. beta2AR [EXT]

#14.#11 OR #12 OR #13

#15. #6 AND #10 AND #14

EXT, Extensive search (mapping, explosion, as keyword)；exp, explosion

Timespan=1966-2014；Date of Search: Jan. 03 .2014

Results: **1059** articles were found

**BIOSIS Previews**

#1.Topic=(polymorphism) OR Topic=(variant*) OR Topic=(genetic*) OR Topic=(mutant*) OR Topic=(single nucleotide)

#2.Topic=(asthma)

#3.Topic=(adrenoceptor) OR Topic=(β2-adrenergic) OR Topic=(beta adrenergic) OR Topic=(ADRB2) OR Topic=(beta2AR)

#4.#3 AND #2 AND #1

Timespan=2000-2014

Date of Search: Jan. 03 .2014

Results: **423** articles were found

- [View results](http://211.103.242.218/GW2/s/com/embase/www/G.http/search/results?viewsearch=23)
- [Edit](http://211.103.242.218/GW2/s/com/embase/www/G.http/search/results)
- [Copy to advanced search](http://211.103.242.218/GW2/s/com/embase/www/G.http/search/advanced?id=23)
- [Set email alert](http://211.103.242.218/GW2/s/com/embase/www/G.http/search/results)
- [Set RSS feed](http://211.103.242.218/GW2/s/com/embase/www/G.http/search/results)
